# A New Approach of Fatigue Classification Based on Data of Tongue and Pulse With Machine Learning

**DOI:** 10.3389/fphys.2021.708742

**Published:** 2022-02-07

**Authors:** Yulin Shi, Xinghua Yao, Jiatuo Xu, Xiaojuan Hu, Liping Tu, Fang Lan, Ji Cui, Longtao Cui, Jingbin Huang, Jun Li, Zijuan Bi, Jiacai Li

**Affiliations:** ^1^Basic Medical College, Shanghai University of Traditional Chinese Medicine, Pudong, China; ^2^Shanghai Innovation Center of TCM Health Service, Shanghai University of Traditional Chinese Medicine, Pudong, China

**Keywords:** fatigue, tongue diagnosis, pulse diagnosis, machine learning, intelligent diagnosis

## Abstract

**Background:**

Fatigue is a common and subjective symptom, which is associated with many diseases and suboptimal health status. A reliable and evidence-based approach is lacking to distinguish disease fatigue and non-disease fatigue. This study aimed to establish a method for early differential diagnosis of fatigue, which can be used to distinguish disease fatigue from non-disease fatigue, and to investigate the feasibility of characterizing fatigue states in a view of tongue and pulse data analysis.

**Methods:**

Tongue and Face Diagnosis Analysis-1 (TFDA-1) instrument and Pulse Diagnosis Analysis-1 (PDA-1) instrument were used to collect tongue and pulse data. Four machine learning models were used to perform classification experiments of disease fatigue vs. non-disease fatigue.

**Results:**

The results showed that all the four classifiers over “Tongue & Pulse” joint data showed better performances than those only over tongue data or only over pulse data. The model accuracy rates based on logistic regression, support vector machine, random forest, and neural network were (85.51 ± 1.87)%, (83.78 ± 4.39)%, (83.27 ± 3.48)% and (85.82 ± 3.01)%, and with Area Under Curve estimates of 0.9160 ± 0.0136, 0.9106 ± 0.0365, 0.8959 ± 0.0254 and 0.9239 ± 0.0174, respectively.

**Conclusion:**

This study proposed and validated an innovative, non-invasive differential diagnosis approach. Results suggest that it is feasible to characterize disease fatigue and non-disease fatigue by using objective tongue data and pulse data.

## Introduction

Fatigue refers to the state that the body cannot endure certain physical intensity with both physiological and pathological manifestation (Chaudhuri and Behan, 2004). Fatigue is subjective uncomfortableness. It can be either mental or physical, and can be of different degrees depending on the health conditions (Persson and Bondke Persson, 2016). Studies have shown that chronic fatigue syndrome (CFS) (Wang et al., 2014; Sandler and Lloyd, 2020), depression (Kim et al., 2019), cancer (Lawrence et al., 2004), and other diseases have obvious fatigue manifestations, and various treatment modalities, such as radiotherapy (Hickok et al., 2005; Dhruva et al., 2010), chemotherapy (Minton et al., 2013), and hormone and biological therapy (Phillips et al., 2013) can aggravate fatigue. Fatigue is one of the most common subjective symptoms of abnormal health state and can be further categorized as disease fatigue and non-disease fatigue. Due to the lack of objective diagnostic tool of fatigue, there is still no reliable and stable evaluation method to distinguish disease fatigue and non-disease fatigue.

Traditional Chinese medicine (TCM) leverages symptoms, physical signs, tongue, and pulse as one of the ways to characterize patient health status. With rapid development in computer science, various machine learning methodologies, such as logistic regression (Bucur et al., 2017; Zhang et al., 2018), support vector machine (SVM) (Li X. et al., 2019), random forest (Ozçift, 2011; Kong and Yu, 2018), convolutional neural network (Shin et al., 2016; Yu et al., 2017), and deep neural network (Ben-Bassat et al., 2018) have been widely applied in the field of medical research. Using artificial intelligence methods in understanding the diagnostic data and syndromes or diseases can help improve the accuracy and precision of diagnosis in an objective and efficient manner. In TCM, fatigue is believed to be related to decline of the whole or local functional state of the human body-the performance of Qi deficiency. Tongue diagnosis and pulse diagnosis are recognized diagnostic methods which are based on overall evaluation of human body; and this is suitable in functional states evaluation, forming important foundation for the evaluation of health status and disease diagnosis. Tongue and pulse manifestations are closely related to heart, lung, spleen, stomach, liver, and kidney functioning, just as the old saying goes: “Tongue reflecting sign of heart,” “The tongue is the external phenology of the spleen and stomach,” “Heart dominating blood and vessel,” “The pulse is the house of blood,” and “Lung connecting all vessels.” Tongue and pulse conditions can reflect the function of Qi, blood, and viscera. Therefore, when fatigue occurs, the changes in functions of the heart, lungs, or other viscera will be reflected in tongue and pulse manifestations. Thus, tongue and pulse conditions can be used to understand the severity and cause of fatigue. Using a large amount of patient level data collected by modern tongue diagnostic or pulse diagnostic instruments, a number of diagnostic models have been developed using machine learning in other disease areas (Wang et al., 2013, 2020; Zhang et al., 2019). Based on modern tongue (Ding et al., 2015; Li W.L. et al., 2019) and pulse diagnosis (Shi et al., 2017; Kung et al., 2020) technology, research on fatigue has made great progress.

Fatigue is an early sign of abnormal health status, which plays a very important role in understanding the health status and early prevention and diagnosis of disease. However, due to lack of objective evidence for fatigue, especially in the early stage of the disease, fatigue is often neglected, which delays diagnosis and timely intervention. A reliable and consistent method to distinguish disease fatigue and non-disease fatigue can effectively assist differentiation of disease fatigue and non-disease fatigue in early diagnosis. This study aims to establish a method for early differential diagnosis of fatigue, to facilitate early diagnosis, prevention, and treatment of disease. This is an interdisciplinary work in which we interpret the scientific rules of disease diagnosis based on objective data of tongue and pulse.

## Materials and Methods

### Study Subjects

**FIGURE 1:**
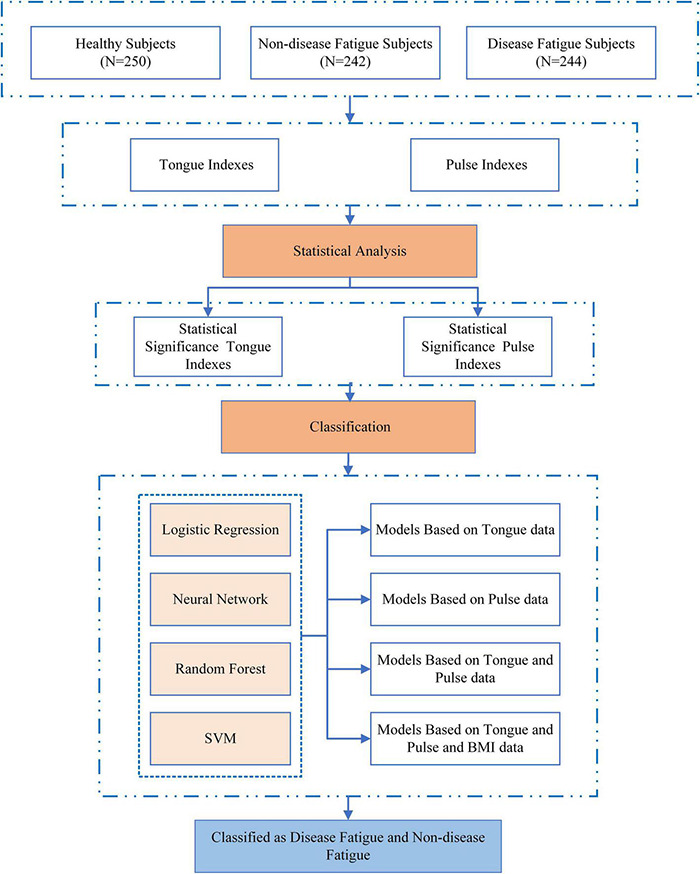
Overall flowchart.

A total of 486 fatigue patients were included in this study from January 2015 to December 2018 at Medical Examination Center of Shuguang Hospital affiliated to Shanghai University of TCM. Patients were divided into two groups by experienced clinicians according to disease diagnostic guidelines and fatigue diagnostic criteria: non-disease fatigue subjects (*n* = 242), and disease fatigue subjects (*n* = 244). The study included a group of healthy population (*n* = 250) as controls. Patient selection and classification is shown in Figure 1. All patients have signed informed consent form.

### Inclusion and Exclusion Criteria

Specific diagnosis of disease for patients with disease fatigue was made by four experienced clinicians following diagnostic criteria of Western medicine. Most common diseases included Chinese Diabetes and Society (2018), Hypertension et al. (2019), and hyperlipidemia (Yan et al., 2017). Health Status Assessment Questionnaire Scale (H20 Scale) and the Information Record Form of Four Diagnosis of TCM (Copyright No.: 2016Z11L025702) (Shi et al., 2021) (as shown in Supplementary Material 1) were used to further define the state of fatigue.

Inclusion criteria: (1) meeting the diagnostic criteria of disease or there are obvious abnormal physical signs for diseases. (2) Have symptom of fatigue.

Exclusion criteria: (1) pregnant or lactating women. (2) Psychopath. (3) Patients with poor compliance.

### Collecting Clinical Tongue Data and Pulse Data

**FIGURE 2:**
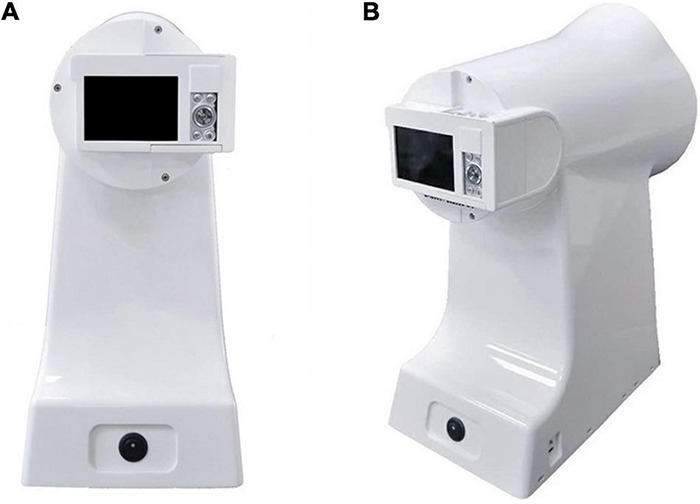
Tongue and Face Diagnosis Analysis-1 (TFDA-1) tongue diagnosis instrument. **(A)** Front view. **(B)** Profile view.

**FIGURE 3:**
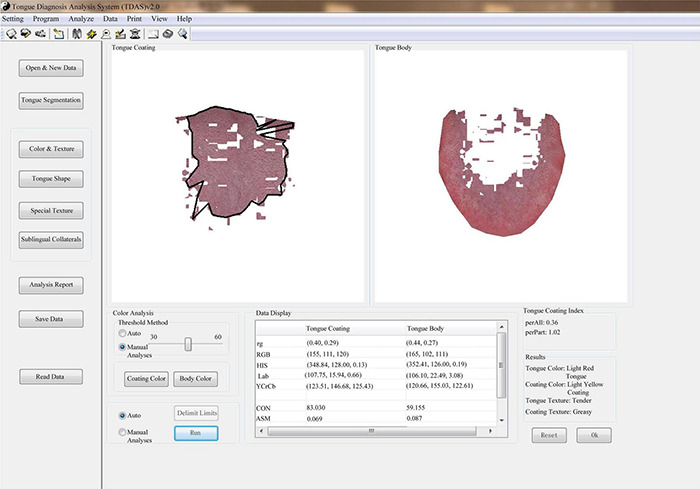
The corresponding software analysis interface of TFDA-1 equipment.

**FIGURE 4:**
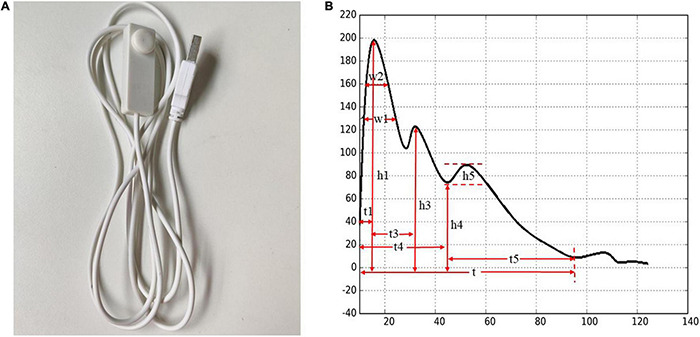
Pulse Diagnosis Analysis-1 (PDA-1) pulse diagnosis instrument and sphygmogram. **(A)** PDA-1 pulse diagnosis instrument. **(B)** Sphygmogram and its parameters.

Tongue and Face Diagnosis Analysis-1 (TFDA-1) instrument and Pulse Diagnosis Analysis-1 (PDA-1) instrument were used to collect tongue data and pulse data. TFDA-1 instrument is shown in Figure 2 and its corresponding analysis software, named tongue diagnosis analysis system (TDAS) V2.0, is shown in Figure 3. The corresponding indices of tongue body and tongue coating could be obtained *via* TDAS. All these indices reflect the tongue characteristics from different perspectives, which served as important objective basis for health status evaluation and syndrome diagnosis. PDA-1 instrument and its corresponding sphygmogram are shown in Figure 4. All investigators were specialized medical students who had been trained for standard study operating procedures to ensure consistency and accuracy of data collection.

The tongue indices were from three color spaces, Lab, HIS, and YCrCb (Qi et al., 2016; Sun et al., 2016; Schiller et al., 2018), each tongue and pulse index had its medical meaning (Qi et al., 2016; Luo et al., 2018; Li et al., 2021b; Shi et al., 2021). The indices of tongue diagnosis and pulse diagnosis and their corresponding clinical meaning are shown in Supplementary Table 1.

### Statistical Analysis

The SPSS 25.0 software was used for statistical analysis. Continuous data with normal distribution are presented as mean and SD, and those with abnormal distribution are presented using median and interquartile range (IQR). Comparisons between groups were conducted using ANOVA or Kruskal–Wallis *H*-test for continuous variables. A *p* < 0.05 (two-tailed) was considered to be statistically significant in comparisons.

### Classification by Machine Learning Approach

In this study, four machine learning methods, such as logistic regression, SVM, neural network, and random forest were used. The random forest is an ensemble learning method for classification and other tasks, which does not utilize the gradient decent. When modeling data by the random forest, no operations of normalizing data were performed. In our experiments by using the three models of logistic regression, SVM, and neural network, the data were normalized using the method of *Z*-score. The preprocessing-data method of *Z*-score is described as the following Eq. 1.


(1) $$Z=\frac{X-\mu }{\sigma }$$

where *X* denotes an element in a data vector, μ for mean value, and σ for SD.

Logistic regression, a multivariate analysis method for studying the relationship between categorical variables and influencing factors, is usually used to construct prediction models for exploring risk factors and predicting the probability of a certain disease. Its accuracy of prediction can be improved by adjusting regression model parameters (Bucur et al., 2017; Zhang et al., 2018). Logistic regression model is described by the following Eq. 2.


(2) $$\text{ln}\frac{\text{y}}{1-\text{y}}=W^{\text{T}}X+\text{b}$$

where *X* denotes a vector for sample, *W* denotes a vector for the linear parameters, and *b* and *y* are scalars.

Support vector machine is one of the most important supervised learning models, used to solve classification or regression problems. Its essence is to find a hyperplane between different data types to create a boundary, which maximizes the interval between data points in different classes. SVM is widely used in face recognition and disease patterns (Li et al., 2012; Zhang et al., 2017).

Random forest is a classifier that uses multiple decision trees to train and predict sample. Though it is not the most accurate classification algorithm, it runs efficiently on large datasets and can handle thousands of input variables without variable deletion (Kong and Yu, 2018). In our random forest, two metrics, i.e., Gini index and information gain, were separately taken as criterion to select partition attributes. The Gini index was calculated according to Eqs 3, 4, and the information gain by using Eqs 5, 6.


(3) $$Gini\left(D\right)=1-\sum_{k=1}^{n}p_{k}^{2}$$

(4) $$Gini\_index\left(D,a\right)=\sum_{v=1}^{v}\frac{\left|D^{v}\right|}{\left|D\right|}Gini\left(D^{v}\right)$$

(5) $$Ent\left(D\right)=-\sum_{k=1}^{n}p_{k}log_{2}p_{k}$$

(6) $$Gain\left(D,a\right)=\text{Ent}\left(\text{D}\right)-\sum_{v=1}^{v}\frac{\left|D^{v}\right|}{\left|D\right|}Ent\left(D^{v}\right)$$

where *D* denotes a data set, *n* for the total number of categories in the data set *D.* Symbol *p_k_* is a probability of a sample being classified to be the *k*-th category. In other words, *p*_*k*_ means a ratio that the *k*-th category accounts for in the dataset. Symbol *a* represents an attribute, *V* for the number of sets obtained by partitioning the set *D* according to the attribute *a*, *D^v^* for a subset of the set *D* corresponding to a value of the attribute *a*.

Neural network is another important machine learning method. It can simulate human brain to achieve artificial intelligence. Our neural network contained one hidden layer with activation function. Three activation functions, such as Tanh, Sigmoid, and ReLU, were selected respectively in the hidden layer. The computation in the hidden layer with activation Tanh is presented in Eq. 7, Eq. 8 is for computations in the type of hidden layer with activation Sigmoid, and Eq. 9 for the type of hidden layer with activation ReLU. Two optimizers, i.e., adaptive moment estimation (Adam) and stochastic gradient decent optimizer (SGD), are taken, respectively.


(7) $$y=\tanh\left(W^{T}\times \text{X}-\theta \right)=\frac{e^{\left(W^{T}\times X-\theta \right)}-e^{-\left(W^{T}\times X-\theta \right)}}{e^{\left(W^{T}\times X-\theta \right)}+e^{-\left(W^{T}\times X-\theta \right)}}$$

(8) $$y=\sigma \left(W^{T}\times \text{X}-\theta \right)=\frac{1}{1+e^{-\left(W^{T}\times X-\theta \right)}}$$

(9) $$y=\text{max}\left(0,W^{T}\times \text{X}-\theta \right)$$

where X is an input vector, *W* for a weight vector, and θ for a threshold.

We used SPSS 25.0 to detect outliers or extreme values of tongue and pulse data, the sample who had outliers or extreme values were deleted. All tongue and pulse data were extracted in batches by specialized tongue and pulse diagnosis analysis software, at the same time, we conducted a manual check of all data to ensure that there was no artificial input errors and missing values. All the experiments were performed in Python 3.6. The metric of area under the curve (AUC) was calculated as an area under the receiver operating characteristic curve (ROC). Accuracy, Precision, Sensitivity, Specificity, and F1 were formally defined in the following Eqs 10–14. The accuracy was defined as a ratio between the number of correctly classified samples and the total number of samples. Precision was defined as a ratio of correctly predicted positive samples out of predicted positive samples. F1-score is the harmonic mean of Precision and Sensitivity (Yang et al., 2018). Sensitivity was defined as the proportion of positive samples which are correctly identified, which measures the ability of classifier to correctly identify positive samples. Specificity is the proportion of negatives which are correctly predicted (Handelman et al., 2018).


(10) $$\text{Accuracy}=\frac{\text{TP}+\text{TN}}{\text{TP}+\text{TN}+\text{FP}+\text{FN}}\times 100\%$$

(11) $$\text{Precision}=\frac{\text{TP}}{\text{TP}+\text{FP}}\times 100$$

(12) $$\text{Sensitivity}=\frac{\text{TP}}{\text{TP}+\text{FN}}\times 100\%$$

(13) $$\text{Specificity}=\frac{\text{TN}}{\text{TN}+\text{FP}}\times 100\%$$

(14) $$F=\frac{2\times \text{Precision}\times \text{Sensitivity}}{\text{Precision}+\text{Sensitivity}}$$

True Positive (TP) is the number of positive samples which are correctly predicted. True Negative (TN) is the number of negative samples which are correctly predicted. False Positive (FP) denotes the number of negative samples which are predicted to be positive. False Negative (FN) is the number of positive samples predicted to be negative.

### Visualization of Machine Learning

Predicted results of machine learning models were visualized by using t-distributed stochastic neighbor embedding (t-SNE). The visualization intuitively showed predicted results and capabilities of machine learning models. The t-SNE algorithm was deployed to reduce the high-dimensional data collected in this study into two-dimensional data. The features in each dimension of the obtained two-dimensional data were rescaled to the range of by using min-max normalization. A general formula for the min-max normalization was given as Eq. 15, where an original value in a dimension was the normalized value. Normalized data were then scattered on a two-dimensional plane.


(15) $$x^{\prime }=\frac{\text{x}-\text{min}\left(\text{x}\right)}{\max\left(x\right)-\text{min}\left(x\right)}$$

## Results

### Basic Statistics

The baseline characteristics of the subjects are presented in Table 1.

**TABLE 1:** Baseline characteristic [median (P25, P75)].

Characteristics	Non-disease fatigue subjects (*n* = 242)	Disease fatigue subjects (*n* = 244)			
		Hypertension and diabetes (*n* = 78)	Hypertension hyperlipemia (*n* = 166)	Diabetes and hyperlipemia (*n* = 87)	Diabetes, hypertension, and hyperlipemia (*n* = 48)
Male/female	146/96	62/16	126/40	72/15	39/9
Age (year)	32.00 (28.00, 37.00)	56.50 (49.75, 65.00)**	50.00 (39.75, 58.00)**##	55.00 (48.00, 64.00)**^⋆^	54.00 (47.25, 64.00)**
BMI (Kg/m^2^)	22.39 (20.28, 24.68)	26.15 (24.08, 28.08)**	25.70 (23.70, 27.40)**	26.10 (24.20, 27.70)**	26.30 (24.68, 27.65)**

vs. Non-disease fatigue subjects, **p < 0.01.

*vs. Hypertension and diabetes, ^##^p < 0.01.*

*vs. Hypertension and hyperlipemia, ^⋆^p < 0.05.*

There were statistically significant differences in age and body mass index (BMI) between disease fatigue and non-disease fatigue group subjects (*p* < 0.01). Patients with disease fatigue who were older are associated with higher BMI.

### Statistical Analysis Over Tongue Data

We selected the widely recognized tongue indices for statistical analysis based on experience from previous studies. The result of tongue indices among three groups are depicted in Table 2. The prefix TB-represents the tongue body, and TC-represents the tongue coating.

**TABLE 2:** Statistical analysis of tongue body and tongue coating index [mean (SD), median (P_25_, P_75_)].

Domain	Color space	Index	Healthy subjects (*n* = 250)	Non-disease fatigue subjects (*n* = 242)	Disease fatigue subjects (*n* = 244)
TB	Lab	TB-L	103.69 (5.37)	103.75 (5.68)	104.84 (6.53)
		TB-a	19.37 (17.57, 21.70)	20.47 (18.10, 22.68)	21.12 (18.69, 23.47)**
		TB-b	6.41 (5.03, 7.80)	5.04 (0.55, 6.93)**	1.71 (−5.33, 5.29)**##
	HIS	TB-H	179.13 (177.00, 181.66)	176.63 (167.87, 180.00)**	170.80 (153.12, 176.89)**##
		TB-S	0.17 (0.15, 0.19)	0.18 (0.15, 0.20)	0.19 (0.16, 0.21)**
		TB-I	117.00 (107.00, 129.00)	118.00 (106.00, 130.00)	120.00 (112.00, 135.00)**#
	YCrCb	TB-Y	114.64 (12.77)	114.98 (13.50)	117.98 (15.76)
		TB-Cr	151.61 (149.13, 153.91)	151.31 (148.38, 153.87)	150.66 (147.88, 153.85)
		TB-Cb	120.28 (118.98, 121.48)	121.31 (119.83, 125.29)**	124.01 (120.70, 130.69)**##
TC	Lab	TC-L	107.91 (103.96, 111.42)	107.93 (104.09, 112.05)	109.14 (105.15, 113.13)*
		TC-a	12.14 (2.52)	12.76 (2.75)*	12.71 (3.03)
		TC-b	4.84 (3.77, 6.20)	3.26 (−1.05, 5.10)**	0.88 (−6.59, 4.08)**##
	HIS	TC-H	181.80 (180.00, 184.84)	177.49 (161.89, 182.42)**	169.76 (132.73, 178.59)**##
		TC-S	0.12 (0.03)	0.12 (0.03)	0.12 (0.03)
		TC-I	126.00 (115.00, 137.00)	127.00 (115.00, 140.00)	131.00 (119.00, 146.00)**#
	YCrCb	TC-Y	122.89 (113.53, 132.11)	122.94 (113.80, 133.72)	125.92 (116.58, 137.03)**
		TC-Cr	143.66 (141.08, 146.02)	143.23 (140.63, 145.77)	142.44 (138.66, 145.91)**
		TC-Cb	121.78 (120.71, 123.11)	123.24 (121.63, 127.73)**	125.61 (122.51, 133.18)**##
	Area index	perAll	0.47 (0.40, 0.60)	0.52 (0.41, 0.76)	0.62 (0.43, 0.90)**#
		perPart	1.14 (1.04, 1.26)	1.09 (1.03, 1.22)	1.05 (1.02, 1.18)**#

vs. Healthy subjects, *p < 0.05, vs. healthy subjects, **p < 0.01.

*vs. Non-disease fatigue subjects, #p < 0.05, vs. non-disease fatigue subjects, ##p < 0.01.*

Statistical results of tongue data showed that TB-a, TB-b, TB-H, TB-S, TB-I, TB-Cb, TC-L, TC-H, TC-I, TC-Y, TC-Cr, TC-Cb, perAll, and perPart showed significant differences among three groups. The numerical distribution trend of the indices of TB-L, TB-a, TB-S, TB-I, TB-Y, TB-Cb, TC-L, TC-I, TC-Y, TC-Cb, and perAll was as follows: healthy subjects < non-disease fatigue subjects < disease fatigue subjects; the numerical distribution trend of the indices of TB-b, TC-b, TB-Cr, TC-Cr, TB-H, TC-H, and perPart had the following order: disease fatigue subjects < non-disease fatigue subjects < healthy subjects.

### Statistical Analysis Over Pulse Data

Similar as in tongue data analysis, the widely used pulse indices were selected for statistical analysis. Results of pulse indices among healthy subjects, non-disease fatigue subjects, and disease fatigue subjects are depicted in Table 3.

**TABLE 3:** Statistical analysis of pulse index [median (P_25_, P_75_)].

Index	Healthy subjects (*n* = 250)	Non-disease fatigue subjects (*n* = 242)	Disease fatigue subjects (*n* = 244)
t_1_(s)	0.13 (0.12, 0.14)	0.13 (0.12, 0.14)	0.14 (0.13, 0.15)**##
t_4_(s)	0.34 (0.33, 0.36)	0.34 (0.33, 0.36)	0.36 (0.34, 0.38)**##
t_5_(s)	0.41 (0.39, 0.42)	0.40 (0.39, 0.42)	0.41 (0.39, 0.43)
t(s)	0.83 (0.77, 0.90)	0.82 (0.77, 0.90)	0.82 (0.75, 0.92)
h_1_(mv)	113.47 (96.27, 135.47)	110.79 (90.78, 132.93)	115.17 (88.77, 146.18)
h_3_(mv)	72.90 (56.40, 90.74)	70.12 (56.00, 87.64)	71.40 (52.46, 104.51)
h_4_(mv)	43.92 (35.09, 53.81)	41.99 (33.05, 51.75)	41.67 (30.19, 56.70)
h_5_(mv)	3.5 (1.13, 6.71)	3.24 (0.65, 6.09)	0.87 (−0.53, 3.16)**##
w_1_(s)	0.17 (0.13, 0.19)	0.17 (0.14, 0.19)	0.18 (0.15, 0.20)**#
w_2_(s)	0.11 (0.09, 0.14)	0.11 (0.09, 0.14)	0.13 (0.11, 0.16)**##
w_1_/t	0.20 (0.17, 0.23)	0.20 (0.18, 0.23)	0.22 (0.19, 0.24)**#
w_2_/t	0.13 (0.11, 0.16)	0.14 (0.11, 0.17)	0.16 (0.13, 0.18)**##

vs. Healthy subjects, **p < 0.01.

*vs. Non-disease fatigue subjects, #p < 0.05, vs. non-disease fatigue subjects, ##p < 0.01.*

Statistical results of pulse indices showed that t_1_, t_4_, h_5_, w_1_, w_2_, w_1_/t, and w_2_/t showed significant difference among three groups (*p* < 0.05 and *p* < 0.01), and the numerical distribution trend of the indices of t_1_, t_4_, w_1_, w_2_, w_1_/t, and w_2_/t was that the group of disease fatigue was larger than the group of non-disease fatigue and the health controls, the numerical distribution trend of h_5_ was as follows: disease fatigue subjects < non-disease fatigue subjects < healthy subjects.

### Results Using Machine Learning and Visualization

Based on the statistical analysis over tongue data and pulse data (Tables 2, 3), such tongue indices and pulse indices showing significant statistic inferences were utilized to characterize disease fatigue and non-disease fatigue. Logistic regression, SVM, random forest, and neural network were deployed as classification models over the datasets, respectively, such as “Tongue,” “Pulse,” “Tongue & Pulse,” and “Tongue & Pulse & Age & BMI.” A dataset in each of our experiments was randomly split into training set and testing set according to a ratio of 8:2. For each of the four models, a procedure of adjusting model parameters was performed separately for each of the four datasets. A setting of parameters with best performances was selected for a model over a dataset. Based on the selected parameters setting, experiments were conducted for 10 times over the corresponding dataset by using the selected model. Classification results of 10 experiments were described in the form of “mean ± SD” for each model over each dataset. They are depicted in Table 4. The results from 10 times repeated modeling of the best parameters of each model are depicted in Supplementary Tables 2–5.

**TABLE 4:** Classification results of disease fatigue against non-disease fatigue over four datasets using four classifiers.

Classifiers	Data sets	Sensitivity (%)	Specificity (%)	F1	Precision (%)	Accuracy (%)	AUC
Logistic regression	Tongue	60.82 ± 4.26	64.49 ± 5.71	0.6192 ± 0.0272	63.32 ± 3.41	62.65 ± 2.67	0.6666 ± 0.0284
	Pulse	62.86 ± 6.88	63.67 ± 4.36	0.6297 ± 0.0476	63.33 ± 3.37	63.27 ± 3.93	0.6990 ± 0.0370
	Tongue & Pulse	67.76 ± 6.11	67.14 ± 6.35	0.6749 ± 0.0457	67.49 ± 4.75	67.45 ± 4.32	0.7395 ± 0.0415
	Tongue & Pulse & Age & BMI	84.90 ± 3.56	86.12 ± 4.36	0.8542 ± 0.0181	86.18 ± 3.67	85.51 ± 1.87	0.9160 ± 0.0136
SVM	Tongue	56.33 ± 4.58	68.78 ± 7.13	0.6004 ± 0.0303	64.71 ± 4.70	62.55 ± 3.10	0.6470 ± 0.0430
	Pulse	64.29 ± 4.76	65.71 ± 3.00	0.6468 ± 0.0313	65.21 ± 2.20	65.00 ± 2.50	0.7035 ± 0.0243
	Tongue & Pulse	65.10 ± 6.42	68.57 ± 5.34	0.6617 ± 0.0506	67.48 ± 4.60	66.84 ± 4.59	0.7203 ± 0.0389
	Tongue & Pulse & Age & BMI	85.31 ± 5.83	82.24 ± 5.56	0.8399 ± 0.0439	82.92 ± 4.78	83.78 ± 4.39	0.9106 ± 0.0365
Random forest	Tongue	61.84 ± 6.89	68.37 ± 4.00	0.6380 ± 0.0521	66.08 ± 4.14	65.10 ± 4.40	0.6803 ± 0.0630
	Pulse	60.61 ± 5.78	61.84 ± 6.89	0.6097 ± 0.0498	61.52 ± 5.39	61.22 ± 5.14	0.6582 ± 0.0509
	Tongue & Pulse	66.94 ± 5.38	70.41 ± 5.79	0.6806 ± 0.0356	69.57 ± 4.22	68.67 ± 3.32	0.7423 ± 0.0444
	Tongue & Pulse & Age & BMI	84.90 ± 4.40	81.63 ± 4.74	0.8353 ± 0.0344	82.33 ± 3.99	83.27 ± 3.48	0.8959 ± 0.0254
Neural network	Tongue	62.45 ± 5.86	63.88 ± 5.78	0.6281 ± 0.0403	63.47 ± 3.72	63.16 ± 3.49	0.6639 ± 0.0255
	Pulse	65.31 ± 7.36	64.90 ± 6.04	0.6500 ± 0.0390	65.17 ± 2.89	65.10 ± 2.77	0.7087 ± 0.0330
	Tongue & Pulse	65.7 ± 12.27	70.2 ± 13.20	0.6664 ± 0.0633	70.34 ± 6.27	67.96 ± 3.33	0.7454 ± 0.0349
	Tongue & Pulse & Age & BMI	85.31 ± 6.57	86.33 ± 1.84	0.8562 ± 0.0353	86.19 ± 1.50	85.82 ± 3.01	0.9239 ± 0.0174

**FIGURE 5:**
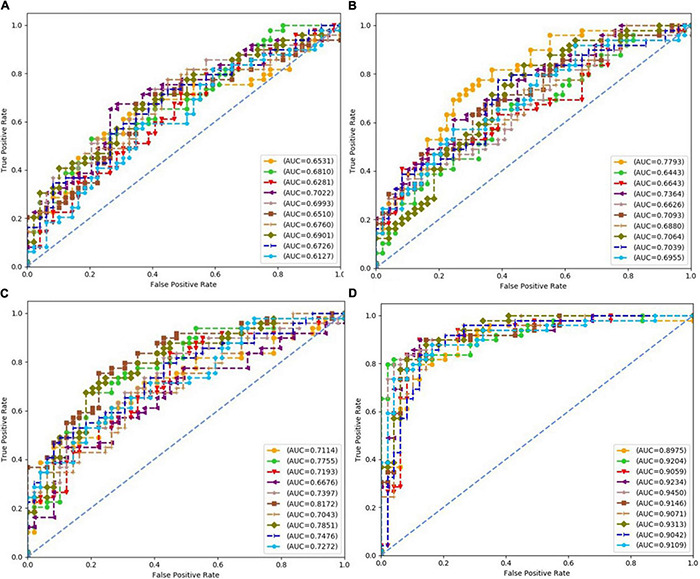
Receiver operating characteristics (ROCs) of 10 times repeated experiments obtained using logistic regression over four datasets. **(A)** ROCs over “Tongue” dataset. **(B)** ROCs over “Pulse” dataset. **(C)** ROCs over “Tongue & Pulse” dataset. **(D)** ROCs over “Tongue & Pulse & Age & BMI” dataset.

**FIGURE 6:**
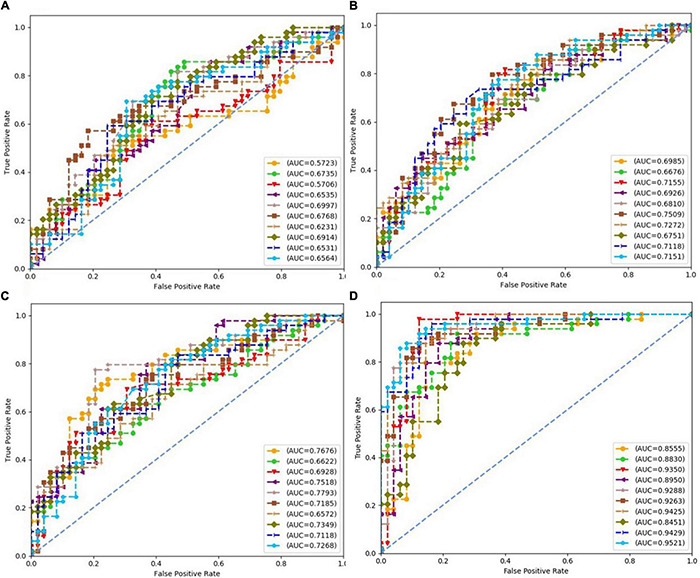
Receiver operating characteristics of 10 times repeated experiments obtained using support vector machine (SVM) over four datasets. **(A)** ROCs over “Tongue” dataset. **(B)** ROCs over “Pulse” dataset. **(C)** ROCs over “Tongue & Pulse” dataset. **(D)** ROCs over “Tongue & Pulse & Age & BMII” dataset.

**FIGURE 7:**
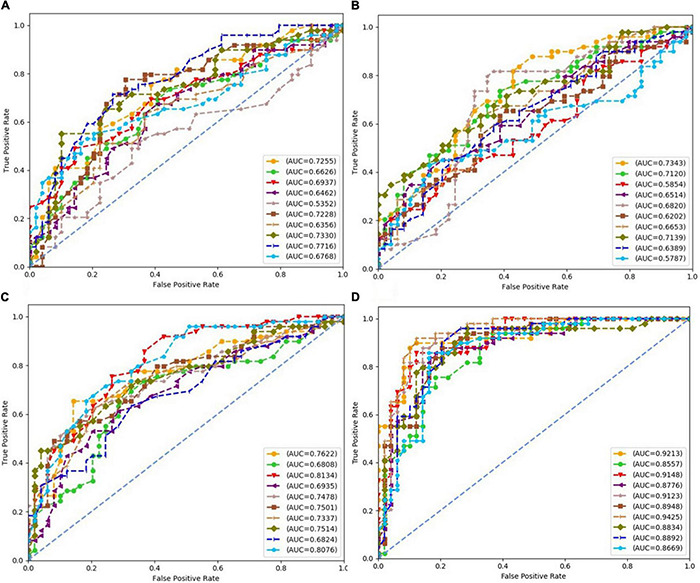
Receiver operating characteristics (ROCs) of 10 times repeated experiments obtained using random forest over four datasets. **(A)** ROCs over “Tongue” dataset. **(B)** ROCs over “Pulse” dataset. **(C)** ROCs over “Tongue & Pulse” dataset. **(D)** ROCs over “Tongue & Pulse & Age & BMI” dataset.

**FIGURE 8:**
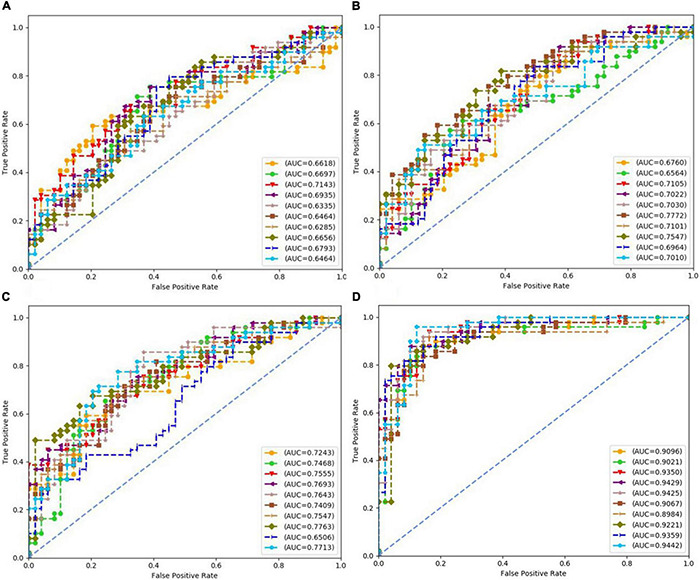
Receiver operating characteristics (ROCs) of 10 times repeated experiments obtained using neural network over four datasets. **(A)** ROCs over “Tongue” dataset. **(B)** ROCs over “Pulse” dataset. **(C)** ROCs over “Tongue & Pulse” dataset. **(D)** ROCs over “Tongue & Pulse & Age & BMI” dataset.

**FIGURE 9:**
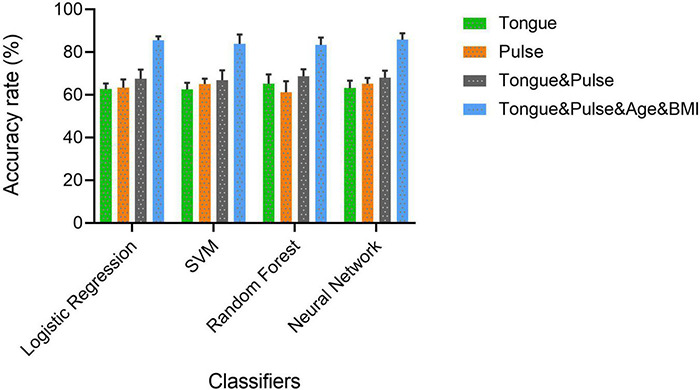
The accuracy rate of four classifiers over four datasets.

Each subfigure in Figures 5–8 plotted 10 ROC curves which were obtained in 10 repeated experiments using a machine model over a dataset, and it gave 10 AUC results corresponding to area under each one of 10 ROC curves. The 10 ROC curves were in different colors, each color represented an ROC result achieved in one experiment. The ROCs of 10 times repeated experiments obtained using logistic regression, SVM, random forest, and neural network over four datasets were depicted in Figures 5–8, respectively. The accuracy rate over four datasets for four machine learning models are depicted in Figure 9.

For all four classifiers, performance over the “Tongue & Pulse” dataset were better than those only using tongue data or pulse data. After adding age and BMI data, the classification efficiency was improved for each of the four models. Over “Tongue & Pulse” dataset, neural network and logistic regression had better classification effects than other classifiers. Overall, the distribution trend of the average accuracy of different classifiers except for random forest based on different datasets had the following order: “Tongue” < “Pulse” < “Tongue & Pulse” < “Tongue & Pulse &Age & BMI.”

**FIGURE 10:**
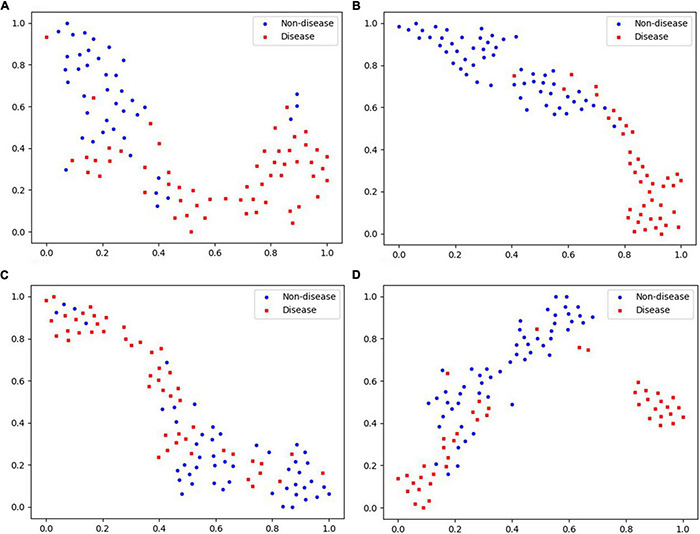
Visualization of “Tongue” data based on different classifiers. **(A)** Logistic regression. **(B)** Neural network. **(C)** Random forest. **(D)** SVM.

**FIGURE 11:**
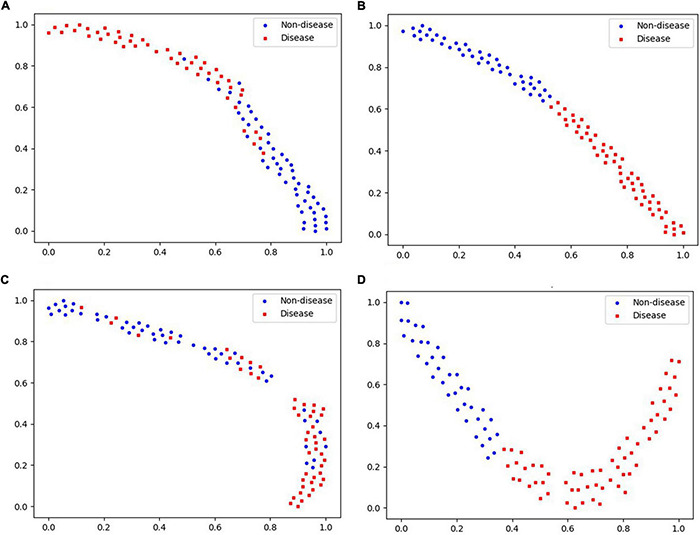
Visualization of “Pulse” data based on different classifiers. **(A)** Logistic regression. **(B)** Neural network. **(C)** Random forest. **(D)** SVM.

There are many different indices of the same diagnosis method, data of a single dimension tends to have a high consistency, so its visualization effect is better. As the data dimension increases, the data complexity increases, and the visualization effect decreases. The visualization of modeling classification results of tongue and pulse sets based on different classifiers in this study are shown in Figures 10, 11. In each subfigure in Figure 10, either blue point or red point represents a two-dimension data point, which was obtained by performing dimensional reduction operation over original testing data and by executing min-max normalization. The abscissa and ordinate were the two dimensions of the two-dimension data obtained by dimensional reduction, respectively.

## Discussion

The purpose of this study was to determine whether general fatigue was caused by diseases and to provide a convenient and reliable method for early screening of fatigue. To achieve this, we enrolled patients undergoing routine physical examination as the research subjects, rather than patients with confirmed disease diagnoses, such as heart disease, cancer, and neurological degenerative diseases, because these patients typically would have definite diagnoses and thus would not meet our research objective to understand early screening for atypical disease fatigue. This study primarily leveraged basic health information and data of tongue and pulse to screen for fatigue population for diseases and non-disease reasons. According to Tables 2, 3, tongue and pulse data of the healthy population overlaps with the two groups of patients with fatigue to a certain extent. The healthy population was selected to serve as baseline to understand general data of tongue and pulse and was not used in modeling for classification.

Our research team has been continuously working on research related to tongue diagnosis technology and has established a relatively reliable analysis methodology for tongue and index, and has also published findings on tongue diagnosis (Zhang et al., 2017; Qi et al., 2018; Qiao et al., 2018; Li et al., 2021a,b; Shi et al., 2021). The index of tongue diagnosis mainly included the color and texture of tongue body and tongue coating and proportion of tongue coating. According to the distribution law of perAll, perPart, TB-Cb, TC-Cb, TB-Cr, TC-Cr, TB-I, TC-I, TB-Y, TC-Y, TB-L, and TC-L, the increase of TC-I, TB-I, TB-L, TC-L, TB-Y, and TC-Y in disease fatigue population indicated white tongue coating, and high perAll and low perPart indicated thick tongue coating. White greasy or white thick coating is generally seen in dampness syndrome or cold syndrome, which were commonly seen in patients with qi deficiency of spleen and stomach or poor transportation function of spleen and stomach (Zhang et al., 2013). The increased TB-Cb and TC-Cb, decreased TB-Cr and TC-Cr indicate purple or more cyanotic tongue body, which is generally seen in qi stagnation and blood stasis syndrome or cold syndrome. Generally speaking, patients with coronary heart disease (Zi et al., 2021), or chronic liver disease (Liu et al., 2003), or vasculitis (Xu et al., 2020), or cancer (Hao et al., 2016), often have purple or more cyanotic tongue body. All the indices were quantified by special TDAS software (TDAS V2.0), and the conclusions were made through statistical analysis. In addition, studies have shown that pulse was closely related to cardiovascular function (Hu et al., 2018; Luo et al., 2018). In our study, the statistical result of pulse indices showed that compared with non-disease fatigue and healthy subjects, disease fatigue subjects had more severe functional decline in left ventricular function, peripheral resistance, aortic compliance, vascular wall elasticity, blood viscosity, and other cardiovascular functions. In addition, pulse was influenced by with these indices.

In the section of modeling using machine learning methods, age and BMI, as recognized prognostic factors, were closely related to diseases. Age and BMI were basic information related to human health, which were closely related to diseases. Studies have shown a correlation between age and the incidence of diseases (Wolff et al., 2020), with the increase in age, the risk of disease gradually increased. Previous studies had shown that BMI (Komatsu et al., 2020) was a key factor of diseases, it played an important role in the diagnostic process. Generally speaking, with the increase of age and BMI, the risk of disease gradually increased. In this study, classification models were constructed over “Age & BMI” datasets, and related experimental results showed that age and BMI had a good classification effect for classifying disease fatigue and non-disease fatigue. However, our focus in this study was that whether data of tongue and pulse or tongue and pulse combined with basic information of age and BMI could distinguish different fatigue states well. For classification models only based on “Age & BMI” datasets and that whether age and BMI had any effect on tongue and pulse, they were not our focus. In conclusion, models based on “Tongue & Pulse” datasets had good classification performances for classifying disease fatigue and non-disease fatigue, and adding age and BMI could help improve the classification performances of models. The classification performances of models over “Tongue & Pulse & Age & BMI” datasets were better than models based on datasets of “Tongue,” “Pulse,” “Tongue & Pulse,” and “Age & BMI,” respectively. Because pulse can reflect cardiovascular function and was closely related to health status. It was convincible that the accurate diagnosis rate of pulse was higher than that of tongue. Therefore, age, BMI, tongue, and pulse were important factors for the fatigue classification model.

## Limitations and Future Work

This study still had some limitations. First, this study mainly focused on tongue and pulse data differences between two “fatigue” groups (disease and non-disease) from a holistic perspective. However, there are a wide range of diseases that require further analysis. Second, the baseline clinical characteristics of the subjects were not comprehensive enough. In the future, narrowing down the research scope of disease, a large-scale and multicenter epidemiological investigation should be combined, and more complete baseline demographic and clinical characteristics data would be useful in further understanding tongue and pulse data for other diseases.

## Data Availability Statement

The datasets generated and analyzed during the current study are not publicly available due to the confidentiality of the data, which is an important component of the National Key Technology R&D Program of the 13th Five-Year Plan (No. 2017YFC1703301) in China, but are available from the corresponding author on reasonable request.

## Ethics Statement

The study protocol was approved by the IRB of Shuguang Hospital affiliated with Shanghai University of TCM (No. 2018-626-55-01). The patients/participants provided their written informed consent to participate in this study. Written informed consent was obtained from the individual(s) for the publication of any potentially identifiable images or data included in this article.

## Author Contributions

YS and JX designed the study. YS and XY wrote the manuscript. XH and LT performed the data analysis. FL, JC, LC, and JH performed the data collection. JuL, ZB, and JiL contributed to the critical discussion and manuscript revision. All authors contributed to the article and approved the submitted version.

## Conflict of Interest

The authors declare that the research was conducted in the absence of any commercial or financial relationships that could be construed as a potential conflict of interest.

## Publisher’s Note

All claims expressed in this article are solely those of the authors and do not necessarily represent those of their affiliated organizations, or those of the publisher, the editors and the reviewers. Any product that may be evaluated in this article, or claim that may be made by its manufacturer, is not guaranteed or endorsed by the publisher.
