# Supplementary material for: A New Approach of Fatigue Classification Based on Data of Tongue and Pulse With Machine Learning
Source: Front Physiol. 2022 Feb 7;12:708742. doi: 10.3389/fphys.2021.708742 (PMC8859319; doi:10.3389/fphys.2021.708742)
Supplement: Supplementary file 1 [file Data_Sheet_1.PDF]

健康状态评估问卷量表（H20量表）

请根据您**最近3个月**的情况，填写下列问题。

1、您觉得自己精力充沛，注意力集中，能正常担负日常生活工作吗？

☐完全是 ☐多数是 ☐一般 ☐很少是 ☐完全不是

2、您感觉到疲劳、乏力，或疲劳以后休息仍不能缓解吗?

☐完全没有 ☐很少有 ☐有 ☐多数有 ☐几乎总是

3、您觉得自己能进行正常社会交往，有良好的人际关系吗？

☐完全是 ☐多数是 ☐一般 ☐很少是 ☐完全不是

4、您觉得自己处事乐观，态度积极吗？

☐完全是 ☐多数是 ☐一般 ☐很少是 ☐完全不是

5、您会出现紧张、焦虑，或急躁、易怒等某些异常情绪吗？

☐完全没有 ☐很少有 ☐有 ☐多数有 ☐几乎总是

6、您容易感冒吗（每年>3次）？

☐完全没有 ☐很少有 ☐有 ☐多数有 ☐几乎总是

7、您有头颈部疼痛、酸胀，或头晕等不适吗？

☐完全没有 ☐很少有 ☐有 ☐多数有 ☐几乎总是

8、您有眼睛酸胀、干涩、疼痛等眼睛不适吗？

☐完全没有 ☐很少有 ☐有 ☐多数有 ☐几乎总是

9、您有耳鸣、或听力减退等耳部不适吗？

☐完全没有 ☐很少有 ☐有 ☐多数有 ☐几乎总是

10、您有口腔溃疡、牙龈肿痛出血、牙根松动等口腔问题吗？

☐完全没有 ☐很少有 ☐有 ☐多数有 ☐几乎总是

11、您有咽喉干燥、痒痛，或有异物感等咽喉不适吗？

☐完全没有 ☐很少有 ☐有 ☐多数有 ☐几乎总是

12、您有咳嗽、气急，或胸闷、心慌吗？

☐完全没有 ☐很少有 ☐有 ☐多数有 ☐几乎总是

13、在没有活动或不是很炎热的情况下，您也很容易出汗吗？

☐完全没有 ☐很少有 ☐有 ☐多数有 ☐几乎总是

14、您有发冷或发热、瘙痒、麻木、疼痛等肢体不适吗？

☐完全没有 ☐很少有 ☐有 ☐多数有 ☐几乎总是

15、您有难以入睡或多梦、易醒等睡眠问题吗？

☐完全没有 ☐很少有 ☐有 ☐多数有 ☐几乎总是

16、您有口中异味，或食欲减退，胃部不适等消化问题吗？

☐完全没有 ☐很少有 ☐有 ☐多数有 ☐几乎总是

17、您有腹泻、便秘等排便问题吗？

☐完全没有 ☐很少有 ☐有 ☐多数有 ☐几乎总是

18、您有尿频、尿急、尿不尽，或夜尿多（≥2次）等排尿问题吗？

☐完全没有 ☐很少有 ☐有 ☐多数有 ☐几乎总是

19、根据您的性别：您有（**女**）月经、白带异常，或（**男**）遗精、早泄等妇科或男科问题吗？

☐完全没有 ☐很少有 ☐有 ☐多数有 ☐几乎总是

20、您对自己的性生活满意吗？

☐很满意（或暂无）☐满意 ☐一般 ☐不满意 ☐很不满意

21、自我评分：请您给自己的总体健康状态评分（满分100分）___________。

中医临床诊断记录表（V2018）

程度较轻或偶尔发生标记为 1；较重或经常发生标记为 2；没有的症状不作任何标记。

精神：疲倦_；抑郁_；喜叹息_；烦躁易怒_；易受惊吓_；多疑善虑_；健忘_；失眠_；多梦_；嗜睡_。

寒热：恶寒_；畏寒肢冷_；低热_；烦热_；烘热_；潮热_；高热_；寒热交替_；喜冷_；喜热_。

问汗：自汗（动则汗出）_；盗汗（夜晚汗出）_；多汗_；无汗_；易感冒_。

头部：头晕_；头重_；听力减退_；耳鸣_；目眩_；目干涩_；视物昏糊_；齿龈肿痛_；齿龈出血_；牙齿松动_；咽喉肿痛_；咽喉梗阻_；咽干_；咽痒_；口腔溃疡_；清涕_；黄涕_；鼻塞_；
鼻痒喷嚏_。

胸腹：乏力_；身重_；腰酸_；胸闷_；心悸_；胃脘闷胀_；胃脘灼热_；胁肋闷胀_；大腹胀_；小腹坠胀_；
腹冷_；肢体麻木_；皮肤瘙痒_；偏瘫_；肠鸣_；矢气_；腹部肿块_；乳房肿块_。

饮食：食少_；多食_；食欲亢进_；多食易饥_；呃逆_；嗳气_；泛酸_；嘈杂_；恶心_；干呕_；呕吐清稀_；
呕吐臭秽_；呕吐痰涎_。

口味：口淡_；口苦_；口甜_；口咸_；粘腻_；口气_；口干_；口渴多饮_；口渴不多饮_。

咳痰：干咳少痰_；咳嗽多痰_；痰清稀_；粘痰_；泡沫_；痰白_；痰黄_；痰中夹血_；脓血痰_。

大便：便秘_；泄泻_；溏薄_；完谷不化_；五更泻_；水样便_；脓血便_；黑便_；溏结不调_；秽臭_；

小便：少尿_；多尿_；余沥不尽_；小便灼热_；夜尿_；尿频_；尿痛_；尿清长_；尿黄_；尿血_；尿浊_；

疼痛：部位（头_；肩颈_；胸_；胁_；胃脘_；大腹_；小腹_；少腹_；腰背_；四肢_）。
性质（酸痛_；胀痛_；闷痛_；刺痛_；冷痛_；灼痛_；游走痛_；隐痛_；绞痛_；喜按_；拒按_；）
程度（偶然_；经常_；持续_；轻微_；剧烈_）。

月经：经期紊乱_；闭经_；痛经_；量多_；量少_；色淡_；色鲜红_；紫暗血块_。

带下：量多_；清稀_；色白_；色黄_；赤白相间_；微臭_；秽臭_。

性功能：性冷淡_；滑精_；早泄_；阳痿_；不孕不育_。

其他：____________。

神识：得神_；少神_；失神_；神乱_；

面色：淡白_；苍白_；满面通红_；颧红_；淡紫_；青紫_；萎黄_；黄疸_；晦暗_；黧黑_；面部痤疮_；

眼目：眼胞色黑_；眼胞肿_；眼球突出_；巩膜黄染_；目眦白_；目红_；

唇：唇色淡白_；唇色红_；唇色淡紫_；唇色青紫_；唇色紫黑_；唇色暗_；口疮_；

甲：甲色淡白_；甲色淡紫_；甲色青紫_；甲色红_；

皮肤：干燥_；湿润_；皮疹_；浮肿_；黄染_；甲错_；粗糙_；黧黑_；多毛_；脱发_；

声息：声高有力_；声低无力_；喘息_；咳喘无力_；上气_；少气_；言语不清_；独语_；错语_；失语_。

舌质：淡红舌_；淡白舌_；红绛舌_；淡紫舌_；青紫舌_；暗红舌_；舌尖红_；舌边红_；
老_；嫩_；胖_；瘦_；点刺_；齿痕_；裂纹_；瘀点_；瘀斑_；溃疡_；舌衄_；
歪斜_；萎软_；僵硬_；吐弄_；震颤_；
舌脉浅淡_；舌脉怒张_；舌脉青紫_；舌脉紫黑_。

舌苔：薄_；白_；黄_；灰_；黑_；少_；剥_；润_；滑_；燥_；糙_；厚_；腻_；腐_。

脉象：

左：浮、沉；迟、数、疾；实、虚；滑、涩；洪、细；长、短；濡、弦、紧；弱、微；结、代、促

右：浮、沉；迟、数、疾；实、虚；滑、涩；洪、细；长、短；濡、弦、紧；弱、微；结、代、促
