# Supplementary material for: A New Approach of Fatigue Classification Based on Data of Tongue and Pulse With Machine Learning
Source: Front Physiol. 2022 Feb 7;12:708742. doi: 10.3389/fphys.2021.708742 (PMC8859319; doi:10.3389/fphys.2021.708742)
Supplement: Supplementary file 2 [file Table_1.DOCX]

Table 1 The indexes of tongue diagnosis and pulse diagnosis and their corresponding clinical meaning

| Features | Meaning |
| --- | --- |
| TB/TC-R | Red, the larger the R value, the redder the color of the tongue body or the thinner the tongue coating |
| TB/TC-G | Green, the larger the G value, the more green components the tongue, tongue body change to be pale |
| TB/TC-B | Blue, the larger the B value, the more blue components the tongue, tongue body change to be blue or purple |
| TB/TC-Y | Brightness, the smaller the Y value, the darker the tongue will be |
| TB/TC-Cr | The difference between the red part of the RGB input signal and the brightness value of the RGB signal |
| TB/TC-Cb | The difference between the blue part of RGB input signal and the brightness value of RGB signal |
| TB/TC-H | Hue. A range of [0, 2π], where the red Angle is 0, the green angle is 2 π /3, and the blue angle is 4 π /3 |
| TB/TC-S | Saturation |
| TB/TC-I | Intensity |
| TB/TC-L | Lightness, [0,100] represents the range from pure black to pure white |
| TB/TC-a | The green-red axis, positive a represent red, negative a represent green |
| TB/TC-b | Blue-yellow axis, positive b represents yellow, negative b represents blue |
| perAll | The ratio of the coating area to the total tongue area |
| perPart | The ratio of the coating area to the non-coating tongue area |
| t_1_ | The time value from the start point to the crest point of the main wave on the pulse graph |
| t_4_ | The time value from the start point to the dicrotic notch on the pulse graph. It Corresponds to left ventricular systolic duration |
| t_5_ | The time value from the dicrotic notch to the end point on the pulse graph. It Corresponds to left ventricular diastolic duration |
| h_1_ | Main wave amplitude. It reflects the compliance of the aorta and the cardiac ejection function of the left ventricular |
| h_3_ | Heavy wave front wave amplitude. It mainly reflects the arterial elasticity and peripheral resistance |
| h_4_ | Dicrotic notch amplitude. It mainly reflects the peripheral vascular resistance and the function of aortic valve closure |
| h_5_ | Gravity wave amplitude. It mainly reflects the elasticity of the aorta and the function of the aortic valve |
| w_1_ | 1/3 height of main wave, the duration of maintaining high intravascular pressure |
| w_2_ | 1/5 height of main wave, the duration of maintaining high intravascular pressure |
| w_1_/t | The ratio of the width of the main wave at its 1/3 height to the entire pulse cycle. It represents the duration of elevated aortic pressure |
| w_2_/t | The ratio of the width of the main wave at its 1/5 height to the entire pulse cycle. It represents the duration of elevated aortic pressure |
| t | One pulsating period, corresponds to one cardiac cycle of the left ventricle |
