# Supplementary material for: A New Approach of Fatigue Classification Based on Data of Tongue and Pulse With Machine Learning
Source: Front Physiol. 2022 Feb 7;12:708742. doi: 10.3389/fphys.2021.708742 (PMC8859319; doi:10.3389/fphys.2021.708742)
Supplement: Supplementary file 3 [file Table_2.DOCX]

Table 2 Ten experiments results of the best parameters of Logistic Regression

| Data sets and parameters of each model | No. | Sensitivity | Specificity | F1 | Precision | Accuracy | AUC |
| --- | --- | --- | --- | --- | --- | --- | --- |
| Tongue_using_scaler_  newton-cg_1 | 1 | 55.10% | 73.47% | 0.6067 | 67.50% | 64.29% | 0.6531 |
|  | 2 | 61.22% | 55.10% | 0.5941 | 57.69% | 58.16% | 0.6281 |
|  | 3 | 63.27% | 73.47% | 0.6667 | 70.45% | 68.37% | 0.7022 |
|  | 4 | 61.22% | 67.35% | 0.6316 | 65.22% | 64.29% | 0.6993 |
|  | 5 | 55.10% | 65.31% | 0.5806 | 61.36% | 60.20% | 0.6510 |
|  | 6 | 65.31% | 59.18% | 0.6337 | 61.54% | 62.24% | 0.6760 |
|  | 7 | 57.14% | 65.31% | 0.5957 | 62.22% | 61.22% | 0.6901 |
|  | 8 | 67.35% | 61.22% | 0.6535 | 63.46% | 64.29% | 0.6726 |
|  | 9 | 57.14% | 65.31% | 0.5957 | 62.22% | 61.22% | 0.6127 |
|  | 10 | 65.31% | 59.18% | 0.6337 | 61.54% | 62.24% | 0.6810 |
| Pulse_using_scaler_  sag_1 | 1 | 77.55% | 67.35% | 0.7379 | 70.37% | 72.45% | 0.7793 |
|  | 2 | 51.02% | 69.39% | 0.5618 | 62.50% | 60.20% | 0.6643 |
|  | 3 | 63.27% | 67.35% | 0.6458 | 65.96% | 65.31% | 0.7364 |
|  | 4 | 61.22% | 57.14% | 0.6000 | 58.82% | 59.18% | 0.6626 |
|  | 5 | 65.31% | 65.31% | 0.6531 | 65.31% | 65.31% | 0.7093 |
|  | 6 | 63.27% | 55.10% | 0.6078 | 58.49% | 59.18% | 0.6880 |
|  | 7 | 69.39% | 63.27% | 0.6733 | 65.38% | 66.33% | 0.7064 |
|  | 8 | 57.14% | 65.31% | 0.5957 | 62.22% | 61.22% | 0.7039 |
|  | 9 | 57.14% | 65.31% | 0.5957 | 62.22% | 61.22% | 0.6955 |
|  | 10 | 63.27% | 61.22% | 0.6263 | 62.00% | 62.24% | 0.6443 |
| Tongue & Pulse_not_using_scaler_saga_0.1 | 1 | 63.27% | 67.35% | 0.6458 | 65.96% | 65.31% | 0.7114 |
|  | 2 | 57.14% | 69.39% | 0.6087 | 65.12% | 63.27% | 0.7193 |
|  | 3 | 61.22% | 61.22% | 0.6122 | 61.22% | 61.22% | 0.6676 |
|  | 4 | 67.35% | 65.31% | 0.6667 | 66.00% | 66.33% | 0.7397 |
|  | 5 | 73.47% | 77.55% | 0.7500 | 76.60% | 75.51% | 0.8172 |
|  | 6 | 63.27% | 69.39% | 0.6526 | 67.39% | 66.33% | 0.7043 |
|  | 7 | 69.39% | 77.55% | 0.7234 | 75.56% | 73.47% | 0.7851 |
|  | 8 | 73.47% | 57.14% | 0.6792 | 63.16% | 65.31% | 0.7476 |
|  | 9 | 71.43% | 61.22% | 0.6796 | 64.81% | 66.33% | 0.7272 |
|  | 10 | 77.55% | 65.31% | 0.7308 | 69.09% | 71.43% | 0.7755 |
| Tongue & Pulse & BMI_not_using_scaler_sag_0.5 | 1 | 73.47% | 83.67% | 0.7742 | 81.82% | 78.57% | 0.8642 |
|  | 2 | 75.51% | 73.47% | 0.7475 | 74.00% | 74.49% | 0.8059 |
|  | 3 | 79.59% | 79.59% | 0.7959 | 79.59% | 79.59% | 0.8413 |
|  | 4 | 73.47% | 73.47% | 0.7347 | 73.47% | 73.47% | 0.7922 |
|  | 5 | 77.55% | 73.47% | 0.7600 | 74.51% | 75.51% | 0.8176 |
|  | 6 | 85.71% | 73.47% | 0.8077 | 76.36% | 79.59% | 0.8401 |
|  | 7 | 77.55% | 79.59% | 0.7835 | 79.17% | 78.57% | 0.8684 |
|  | 8 | 79.59% | 83.67% | 0.8125 | 82.98% | 81.63% | 0.8892 |
|  | 9 | 75.51% | 81.63% | 0.7789 | 80.43% | 78.57% | 0.8292 |
|  | 10 | 77.55% | 71.43% | 0.7525 | 73.08% | 74.49% | 0.8517 |
