# Supplementary material for: A New Approach of Fatigue Classification Based on Data of Tongue and Pulse With Machine Learning
Source: Front Physiol. 2022 Feb 7;12:708742. doi: 10.3389/fphys.2021.708742 (PMC8859319; doi:10.3389/fphys.2021.708742)
Supplement: Supplementary file 4 [file Table_3.DOCX]

Table 3 Ten experiments results of the best parameters of Random Forest

| Data sets and parameters of each model | NO. | Sensitivity | Specificity | F1 | Precision | Accuracy | AUC |
| --- | --- | --- | --- | --- | --- | --- | --- |
| Tongue_70_gini_log2 | 1 | 59.18% | 73.47% | 0.6374 | 69.05% | 66.33% | 0.7255 |
|  | 2 | 55.10% | 71.43% | 0.6000 | 65.85% | 63.27% | 0.6937 |
|  | 3 | 63.27% | 63.27% | 0.6327 | 63.27% | 63.27% | 0.6462 |
|  | 4 | 53.06% | 63.27% | 0.5591 | 59.09% | 58.16% | 0.5352 |
|  | 5 | 77.55% | 69.39% | 0.7451 | 71.70% | 73.47% | 0.7228 |
|  | 6 | 63.27% | 63.27% | 0.6327 | 63.27% | 63.27% | 0.6356 |
|  | 7 | 65.31% | 71.43% | 0.6737 | 69.57% | 68.37% | 0.7330 |
|  | 8 | 67.35% | 73.47% | 0.6947 | 71.74% | 70.41% | 0.7716 |
|  | 9 | 59.18% | 69.39% | 0.6237 | 65.91% | 64.29% | 0.6768 |
|  | 10 | 55.10% | 65.31% | 0.5806 | 61.36% | 60.20% | 0.6626 |
| Pulse_100_gini_log2 | 1 | 59.18% | 71.43% | 0.6304 | 67.44% | 65.31% | 0.7343 |
|  | 2 | 55.10% | 51.02% | 0.5400 | 52.94% | 53.06% | 0.5854 |
|  | 3 | 59.18% | 55.10% | 0.5800 | 56.86% | 57.14% | 0.6514 |
|  | 4 | 73.47% | 65.31% | 0.7059 | 67.92% | 69.39% | 0.6820 |
|  | 5 | 61.22% | 61.22% | 0.6122 | 61.22% | 61.22% | 0.6202 |
|  | 6 | 67.35% | 59.18% | 0.6471 | 62.26% | 63.27% | 0.6653 |
|  | 7 | 57.14% | 71.43% | 0.6154 | 66.67% | 64.29% | 0.7139 |
|  | 8 | 57.14% | 63.27% | 0.5895 | 60.87% | 60.20% | 0.6389 |
|  | 9 | 53.06% | 53.06% | 0.5306 | 53.06% | 53.06% | 0.5787 |
|  | 10 | 63.27% | 67.35% | 0.6458 | 65.96% | 65.31% | 0.7120 |
| Tongue & Pulse_60_entropy_sqrt | 1 | 73.47% | 67.35% | 0.7129 | 69.23% | 70.41% | 0.7622 |
|  | 2 | 75.51% | 73.47% | 0.7475 | 74.00% | 74.49% | 0.8134 |
|  | 3 | 63.27% | 65.31% | 0.6392 | 64.58% | 64.29% | 0.6935 |
|  | 4 | 67.35% | 67.35% | 0.6735 | 67.35% | 67.35% | 0.7478 |
|  | 5 | 65.31% | 67.35% | 0.6598 | 66.67% | 66.33% | 0.7501 |
|  | 6 | 73.47% | 67.35% | 0.7129 | 69.23% | 70.41% | 0.7337 |
|  | 7 | 57.14% | 79.59% | 0.6437 | 73.68% | 68.37% | 0.7514 |
|  | 8 | 65.31% | 63.27% | 0.6465 | 64.00% | 64.29% | 0.6824 |
|  | 9 | 65.31% | 81.63% | 0.7111 | 78.05% | 73.47% | 0.8076 |
|  | 10 | 63.27% | 71.43% | 0.6596 | 68.89% | 67.35% | 0.6808 |
| Tongue & Pulse & BMI_90_entropy_sqrt | 1 | 79.59% | 71.43% | 0.7647 | 73.58% | 75.51% | 0.8490 |
|  | 2 | 81.63% | 65.31% | 0.7547 | 70.18% | 73.47% | 0.8261 |
|  | 3 | 73.47% | 65.31% | 0.7059 | 67.92% | 69.39% | 0.7595 |
|  | 4 | 71.43% | 81.63% | 0.7527 | 79.55% | 76.53% | 0.8421 |
|  | 5 | 81.63% | 73.47% | 0.7843 | 75.47% | 77.55% | 0.8309 |
|  | 6 | 71.43% | 71.43% | 0.7143 | 71.43% | 71.43% | 0.7782 |
|  | 7 | 65.31% | 75.51% | 0.6882 | 72.73% | 70.41% | 0.7722 |
|  | 8 | 77.55% | 71.43% | 0.7525 | 73.08% | 74.49% | 0.8172 |
|  | 9 | 75.51% | 79.59% | 0.7708 | 78.72% | 77.55% | 0.8367 |
|  | 10 | 75.51% | 67.35% | 0.7255 | 69.81% | 71.43% | 0.8276 |
