# Supplementary material for: A New Approach of Fatigue Classification Based on Data of Tongue and Pulse With Machine Learning
Source: Front Physiol. 2022 Feb 7;12:708742. doi: 10.3389/fphys.2021.708742 (PMC8859319; doi:10.3389/fphys.2021.708742)
Supplement: Supplementary file 5 [file Table_4.DOCX]

Table 4 Ten experiments results of the best parameters of Neural Network

| Data sets and parameters of each model | No. | Sensitivity | Specificity | F1 | Precision | Accuracy | AUC |
| --- | --- | --- | --- | --- | --- | --- | --- |
| Tongue_using_scaler_adam_160_relu_128_constant_0.0007 | 1 | 63.27% | 73.47% | 0.6667 | 70.45% | 68.37% | 0.6618 |
|  | 2 | 67.35% | 67.35% | 0.6735 | 67.35% | 67.35% | 0.7143 |
|  | 3 | 69.39% | 63.27% | 0.6733 | 65.38% | 66.33% | 0.6935 |
|  | 4 | 63.27% | 55.10% | 0.6078 | 58.49% | 59.18% | 0.6335 |
|  | 5 | 55.10% | 65.31% | 0.5806 | 61.36% | 60.20% | 0.6464 |
|  | 6 | 63.27% | 57.14% | 0.6139 | 59.62% | 60.20% | 0.6285 |
|  | 7 | 51.02% | 71.43% | 0.5682 | 64.10% | 61.22% | 0.6656 |
|  | 8 | 57.14% | 63.27% | 0.5895 | 60.87% | 60.20% | 0.6793 |
|  | 9 | 65.31% | 57.14% | 0.6275 | 60.38% | 61.22% | 0.6464 |
|  | 10 | 69.39% | 65.31% | 0.6800 | 66.67% | 67.35% | 0.6697 |
| Pulse_using_scaler_adam_220_tanh_128_constant_  0.0003 | 1 | 71.43% | 55.10% | 0.6604 | 61.40% | 63.27% | 0.6760 |
|  | 2 | 65.31% | 61.22% | 0.6400 | 62.75% | 63.27% | 0.7105 |
|  | 3 | 67.35% | 65.31% | 0.6667 | 66.00% | 66.33% | 0.7022 |
|  | 4 | 59.18% | 71.43% | 0.6304 | 67.44% | 65.31% | 0.7030 |
|  | 5 | 79.59% | 59.18% | 0.7222 | 66.10% | 69.39% | 0.7772 |
|  | 6 | 55.10% | 69.39% | 0.5934 | 64.29% | 62.24% | 0.7101 |
|  | 7 | 69.39% | 69.39% | 0.6939 | 69.39% | 69.39% | 0.7547 |
|  | 8 | 55.10% | 67.35% | 0.5870 | 62.79% | 61.22% | 0.6964 |
|  | 9 | 69.39% | 57.14% | 0.6538 | 61.82% | 63.27% | 0.7010 |
|  | 10 | 61.22% | 73.47% | 0.6522 | 69.77% | 67.35% | 0.6564 |
| Tongue & Pulse_using_scaler_adam_100_logistic_128_constant_0.007 | 1 | 69.39% | 63.27% | 0.6733 | 65.38% | 66.33% | 0.7243 |
|  | 2 | 63.27% | 71.43% | 0.6596 | 68.89% | 67.35% | 0.7555 |
|  | 3 | 73.47% | 67.35% | 0.7129 | 69.23% | 70.41% | 0.7693 |
|  | 4 | 34.69% | 91.84% | 0.4857 | 80.95% | 63.27% | 0.7643 |
|  | 5 | 71.43% | 69.39% | 0.7071 | 70.00% | 70.41% | 0.7409 |
|  | 6 | 67.35% | 73.47% | 0.6947 | 71.74% | 70.41% | 0.7547 |
|  | 7 | 63.27% | 81.63% | 0.6966 | 77.50% | 72.45% | 0.7763 |
|  | 8 | 81.63% | 40.82% | 0.6780 | 57.97% | 61.22% | 0.6506 |
|  | 9 | 57.14% | 81.63% | 0.6512 | 75.68% | 69.39% | 0.7713 |
|  | 10 | 75.51% | 61.22% | 0.7048 | 66.07% | 68.37% | 0.7468 |
| Tongue & Pulse & BMI_using_scaler_sgd_140_logistic_32_adaptive_  0.0007 | 1 | 67.35% | 81.63% | 0.7253 | 78.57% | 74.49% | 0.8338 |
|  | 2 | 77.55% | 77.55% | 0.7755 | 77.55% | 77.55% | 0.8305 |
|  | 3 | 73.47% | 77.55% | 0.7500 | 76.60% | 75.51% | 0.8372 |
|  | 4 | 79.59% | 79.59% | 0.7959 | 79.59% | 79.59% | 0.8626 |
|  | 5 | 79.59% | 83.67% | 0.8125 | 82.98% | 81.63% | 0.8909 |
|  | 6 | 77.55% | 85.71% | 0.8085 | 84.44% | 81.63% | 0.8655 |
|  | 7 | 75.51% | 77.55% | 0.7629 | 77.08% | 76.53% | 0.8167 |
|  | 8 | 69.39% | 75.51% | 0.7158 | 73.91% | 72.45% | 0.8038 |
|  | 9 | 81.63% | 79.59% | 0.8081 | 80.00% | 80.61% | 0.8959 |
|  | 10 | 65.31% | 79.59% | 0.7033 | 76.19% | 72.45% | 0.8567 |
