# Supplementary material for: A New Approach of Fatigue Classification Based on Data of Tongue and Pulse With Machine Learning
Source: Front Physiol. 2022 Feb 7;12:708742. doi: 10.3389/fphys.2021.708742 (PMC8859319; doi:10.3389/fphys.2021.708742)
Supplement: Supplementary file 6 [file Table_5.DOCX]

Table 5 Ten experiments results of the best parameters of SVM

| Data sets and parameters of each model | No. | Sensitivity | Specificity | F1 | Precision | Accuracy | AUC |
| --- | --- | --- | --- | --- | --- | --- | --- |
| Tongue_using_scaler_rbf_4_3_auto | 1 | 53.06% | 67.35% | 0.5714 | 61.90% | 60.20% | 0.5723 |
|  | 2 | 51.02% | 65.31% | 0.5495 | 59.52% | 58.16% | 0.5706 |
|  | 3 | 65.31% | 53.06% | 0.6154 | 58.18% | 59.18% | 0.6535 |
|  | 4 | 51.02% | 73.47% | 0.5747 | 65.79% | 62.24% | 0.6997 |
|  | 5 | 57.14% | 81.63% | 0.6512 | 75.68% | 69.39% | 0.6768 |
|  | 6 | 55.10% | 69.39% | 0.5934 | 64.29% | 62.24% | 0.6231 |
|  | 7 | 61.22% | 65.31% | 0.6250 | 63.83% | 63.27% | 0.6914 |
|  | 8 | 53.06% | 75.51% | 0.5977 | 68.42% | 64.29% | 0.6531 |
|  | 9 | 61.22% | 69.39% | 0.6383 | 66.67% | 65.31% | 0.6564 |
|  | 10 | 55.10% | 67.35% | 0.5870 | 62.79% | 61.22% | 0.6735 |
| Pulse_using_scaler_rbf_3_3_auto | 1 | 67.35% | 63.27% | 0.6600 | 64.71% | 65.31% | 0.6985 |
|  | 2 | 67.35% | 63.27% | 0.6600 | 64.71% | 65.31% | 0.7155 |
|  | 3 | 63.27% | 61.22% | 0.6263 | 62.00% | 62.24% | 0.6926 |
|  | 4 | 63.27% | 63.27% | 0.6327 | 63.27% | 63.27% | 0.6810 |
|  | 5 | 69.39% | 67.35% | 0.6869 | 68.00% | 68.37% | 0.7509 |
|  | 6 | 59.18% | 69.39% | 0.6237 | 65.91% | 64.29% | 0.7272 |
|  | 7 | 61.22% | 63.27% | 0.6186 | 62.50% | 62.24% | 0.6751 |
|  | 8 | 73.47% | 67.35% | 0.7129 | 69.23% | 70.41% | 0.7118 |
|  | 9 | 57.14% | 69.39% | 0.6087 | 65.12% | 63.27% | 0.7151 |
|  | 10 | 61.22% | 69.39% | 0.6383 | 66.67% | 65.31% | 0.6676 |
| Tongue & Pulse_using_scaler_rbf_3_3_auto | 1 | 73.47% | 67.35% | 0.7129 | 69.23% | 70.41% | 0.7676 |
|  | 2 | 69.39% | 69.39% | 0.6939 | 69.39% | 69.39% | 0.6928 |
|  | 3 | 63.27% | 65.31% | 0.6392 | 64.58% | 64.29% | 0.7518 |
|  | 4 | 77.55% | 79.59% | 0.7835 | 79.17% | 78.57% | 0.7793 |
|  | 5 | 61.22% | 67.35% | 0.6316 | 65.22% | 64.29% | 0.7185 |
|  | 6 | 65.31% | 61.22% | 0.6400 | 62.75% | 63.27% | 0.6572 |
|  | 7 | 65.31% | 65.31% | 0.6531 | 65.31% | 65.31% | 0.7349 |
|  | 8 | 59.18% | 71.43% | 0.6304 | 67.44% | 65.31% | 0.7118 |
|  | 9 | 55.10% | 75.51% | 0.6136 | 69.23% | 65.31% | 0.7268 |
|  | 10 | 61.22% | 63.27% | 0.6186 | 62.50% | 62.24% | 0.6622 |
| Tongue & Pulse & BMI_using_scaler_rbf_2_3_scale | 1 | 71.43% | 79.59% | 0.7447 | 77.78% | 75.51% | 0.8159 |
|  | 2 | 69.39% | 81.63% | 0.7391 | 79.07% | 75.51% | 0.8409 |
|  | 3 | 69.39% | 71.43% | 0.7010 | 70.83% | 70.41% | 0.7430 |
|  | 4 | 77.55% | 79.59% | 0.7835 | 79.17% | 78.57% | 0.8676 |
|  | 5 | 67.35% | 71.43% | 0.6875 | 70.21% | 69.39% | 0.7978 |
|  | 6 | 67.35% | 71.43% | 0.6875 | 70.21% | 69.39% | 0.7609 |
|  | 7 | 75.51% | 71.43% | 0.7400 | 72.55% | 73.47% | 0.7584 |
|  | 8 | 67.35% | 73.47% | 0.6947 | 71.74% | 70.41% | 0.7580 |
|  | 9 | 69.39% | 67.35% | 0.6869 | 68.00% | 68.37% | 0.8055 |
|  | 10 | 79.59% | 77.55% | 0.7879 | 78.00% | 78.57% | 0.8222 |
